# Gender Differences in Sleep Deprivation Effects on Risk and Inequality Aversion: Evidence from an Economic Experiment

**DOI:** 10.1371/journal.pone.0120029

**Published:** 2015-03-20

**Authors:** Michele Ferrara, Anna Bottasso, Daniela Tempesta, Marika Carrieri, Luigi De Gennaro, Giovanni Ponti

**Affiliations:** 1 Department of Life, Health and Environmental Sciences, University of L’Aquila, L’Aquila, Italy; 2 Department of Economics, University of Genova, Genova, Italy; 3 Department of Psychology, Sapienza University of Rome, Rome, Italy; 4 Department of Economics and Finance, LUISS Guido Carli, Rome, Italy; 5 Departamento de Fundamentos del Análisis Económico, Universidad de Alicante, Alicante, Spain; University of Pennsylvania, UNITED STATES

## Abstract

Excessive working hours—even at night—are becoming increasingly common in our modern 24/7 society. The prefrontal cortex (PFC) is particularly vulnerable to the effects of sleep loss and, consequently, the specific behaviors subserved by the functional integrity of the PFC, such as risk-taking and pro-social behavior, may be affected significantly. This paper seeks to assess the effects of one night of sleep deprivation on subjects’ risk and social preferences, which are probably the most explored behavioral domains in the tradition of Experimental Economics. This novel cross-over study employs thirty-two university students (gender-balanced) participating to 2 counterbalanced laboratory sessions in which they perform standard risk and social preference elicitation protocols. One session was after one night of undisturbed sleep at home, and the other was after one night of sleep deprivation in the laboratory. Sleep deprivation causes increased sleepiness and decreased alertness in all subjects. After sleep loss males make riskier decisions compared to the rested condition, while females do the opposite. Females likewise show decreased inequity aversion after sleep deprivation. As for the relationship between cognitive ability and economic decisions, sleep deprived individuals with higher cognitive reflection show lower risk aversion and more altruistic behavior. These results show that one night of sleep deprivation alters economic behavior in a gender-sensitive way. Females’ reaction to sleep deprivation, characterized by reduced risky choices and increased egoism compared to males, may be related to intrinsic psychological gender differences, such as in the way men and women weigh up probabilities in their decision-making, and/or to the different neurofunctional substrate of their decision-making.

## Introduction

Our modern 24/7 society is characterized by the need to work around the clock, resulting in excessive working hours and increasingly longer night shifts. For instance, international financial market professionals often need to work through the night, which hinders optimal decision-taking throughout the trading session. Specifically, if lack of sleep alters the perception of uncertainty associated with a given situation, they may prefer safer investments and avoid risky trades [1]; otherwise, if sleep loss leads to overconfidence, they may believe that they are more likely to succeed in trades and prefer to take greater investment risks [2].

Functional neuroimaging studies on risky decision-making during sleep deprivation have shown that the prefrontal cortex is the linchpin for carrying out many of these higher order and more complex cognitive processes [2, 3]. These studies have likewise pointed out that the prefrontal cortex is particularly vulnerable to the effects of sleep loss and shows widely decreased activation [4]. This effect has been related to the extensive use of the prefrontal cortex during waking hours [5]. As a consequence, the specific behaviors subserved by the functional integrity of the prefrontal cortex, such as risk-taking, are particularly affected by sleep loss [6, 7]. The link between sleep deprivation and risk attitudes has indeed received some attention in the specialized literature. In this respect, both clinical and laboratory data indicate that fatigue and sleep loss are associated with decreased attention and increased risk taking. Killgore et al. [6] show that, after 75 hours of wakefulness, risk-taking as measured by the Balloon Analog Risk Task increases in healthy subjects [6]. Moreover, Harrison and Horne [8], by way of a market simulation game, report that 36 hours of total sleep deprivation lead to stereotyped decisions which fail to integrate previous feedback, resulting in large financial losses and production errors [8]. McKenna et al. [7], by way of simple binary decisions involving lotteries over gains and losses, show that, following as little as 23 hours of sleep deprivation, sleep-deprived subjects are less risk averse for gains and less risk seeking for losses. In other words, sleep deprivation moves behavior in the opposite direction with respect to the classic assumptions of Prospect Theory [9].

Moving on to economic experiments, many studies investigate the existence of gender differences in risk attitudes. In this respect, the debate is far from settled, in that there is evidence suggesting that women engage in less risky behavior [10, 11], while other studies report no significant gender differences in risky behavior [12, 13]. Neuroimaging studies have shown that gender-related differences during risk-taking tasks, when present, are associated to different brain activity in the prefrontal cortex [14]. For instance, men show greater activation in a large area of the right lateral orbitofrontal cortex (OFC) during their performance on the Iowa Gambling Task. In contrast, women have greater activation in the left dorsolateral prefrontal cortex (DLPFC), left medial frontal gyrus and temporal lobe during this task. Similarly, some differences in regional brain activity between males and females have further been found as a function of sleep deprivation [15, 16]. In fact, males show significantly higher activity during sleep loss than females in the left cerebellum posterior lobe, left parietal lobe, and bilateral frontal lobes [16].

Although several studies have explored the relationship between sleep deprivation and risk taking, gender has not been usually taken into account as a possible moderating variable. In fact, there is scarce evidence of a gender effect on risk-taking behavior after sleep deprivation. Acheson et al. (2007) find that sleep loss decreases impulsive behavior with the Balloon Analogue Risk Task in women, but not in men [17]. On the other hand, Chaumet et al. (2009) report an increase of impulsiveness in both men and women after 36 h of extended wakefulness [1].

As far as social preferences are concerned, an increasing amount of experimental literature has been exploring the external factors that affect subjects’ willingness to give or, more generally, their distributional concerns in decisions that affect the welfare of others. In recent years, a large number of Dictator Game (DG) experiments have highlighted several factors as determinants of giving, such as *i) framing effects*, that is, the way in which the Dictator’s decision problem is presented to subjects [18, 19] or *ii) social distance effects*, that is, the degree of social proximity of the Dictator-Recipient relationship [20, 21]. However, the effects of sleep deprivation on social preferences have never been addressed.

As for the relation between social preferences and cognitive abilities, Chen et al. [22], find that subjects who perform better on the Math portion of the Scholastic Aptitude Test (SAT) are more generous in both the Dictator game and in a series of small-stakes “dictatorial” (i.e., unilateral) decisions, known as Social Value Orientation (SVO). This evidence is in line with Ben-Ner et al. [23], who find that a higher performance in the Wonderlic test negatively affects giving, although that contrasts with the recent findings of Benjamin et al. [24], where it is found that school test scores do not affect the Dictator’s giving.

As for gender differences in social preferences, Eckel and Grossman [10] show that women give almost twice as much as men to their paired recipient in the Dictator Game. Andreoni and Vesterlund [25], manipulating the cost-benefit ratio of giving money to the recipient, find that women are more concerned with equalizing payoffs while men are more concerned with efficiency.

The self- and other-oriented rewards on a common scale are associated with the activation inventromedial prefrontal cortex (vmPFC) [26]. Consistently, patients with lesions in this area as well as primary psychopats, who are known to exhibit deficits in empathy and guilt, offer abnormally low amounts in the DG [27]. It has been likewise reported that the decision to act prosocially engages the orbitofrontal cortex [28], a region likewise activated when subjects distribute money equitably [29]. On the other hand, inequitable decision-making is accompanied by the engagement of the anterior insula, a region previously associated with subjective disutility [30]. Therefore, the brain mechanisms involved in experiencing the emotional and social states of self and others may drive egalitarian behaviors. According to this view, it has been recently shown that activity in the dorsomedial prefrontal cortex, a region involved in understanding others’ mental states, predicts both monetary donations to others and time spent helping others [31]. Consequently, given that prosocial behavior is based on the correct functioning of parts of the prefrontal/orbitofrontal cortex, and that large portions of the PFC show largely decreased activation during sleep loss [4], it follows that sleep loss should influence prosocial behavior by possibly reducing it. However, to the best of our knowledge the specific effects of sleep deprivation on inequality aversion have never been previously investigated.

Therefore, bearing in mind that sleep plays a fundamental role in prefrontal cortex functioning and, consequently, in maintaining optimal executive performance effectiveness, we test our working hypothesis that lack of sleep may directly affect *risk* and *social* preferences. By the term “risk preferences” we mean subjects’ attitudes in choice environments characterized by “lotteries”, i.e., (objectively known) probability distributions over a fixed set of monetary prizes; by “social preferences” we refer to subjects’ attitudes over choice environments characterized by “payoff externalities”, i.e., choices which have monetary consequences on others. Precisely, we here employ two of the most popular risk and social preference elicitation protocols in Experimental Economics—Random Lottery Pair (RLP) of Hey and Orme [32] and the Dictator Game of Forsythe et al. [33]—in a within-subject study whose main objective is to measure the impact of sleep deprivation on subjects’ performance. Moreover, we likewise analyze the existence of differential effects of sleep loss on risk taking and altruistic behavior as a function of subjects’ gender and cognitive abilities (measured by Frederick’s [34] classic Cognitive Reflection Test, CRT).

## Methods

### Ethics Statement

The protocol was approved by the Ethics Review Committee of the University of L'Aquila and was conducted in accordance with the Declaration of Helsinki, with explicit written consent obtained from each subject.

### Participants

The experiment was conducted at the Laboratory of Sleep Psychophysiology and Cognitive Neurosciences, Department of Life, Health and Environmental Sciences of the University of L'Aquila.

Thirty-two participants (16 females, 16 males; mean age±SD: 24±2.2 years; age range 20–28 years) were recruited through advertisements in the University of l’Aquila buildings.

Subjects were selected if they had no history of pathological gambling, medical, neurological or psychiatric disorders, nor of medication or drug intake, as assessed by self-reported medical history and by a clinical interview (Structured Clinical Interview for DSM–IV Axis I disorders—SCID 1), [35].

Other inclusion criteria were: habitual sleep duration of 7–8 hours per night, bedtime at midnight ± 1 hour, no nap habit during the day. Subjective measures of participants’ usual sleep were collected by a one-week sleep log, including the experimental days. Subjects were excluded if they scored 10 or higher on the Beck Depression Inventory (BDI) [36] and above 5 on the Pittsburgh Sleep Quality Index (PSQI, Italian version) [37].

During the screening phase, participants were likewise asked to complete Frederick’s [34] Cognitive Reflection Test (CRT), a three-item test designed to measure the tendency to override a prepotent response alternative that is incorrect and to engage in further reflection that leads to the correct response. This test is composed of three questions, as follows:

- CRT_1_. A bat and a ball cost $1.10. The bat costs $1.00 more than the ball. How much does the ball cost? ___ cents. **Correct Answer: 5**.
- CRT_2_. If it takes 5 machines 5 minutes to make 5 widgets, how long would it take 100 machines to make 100 widgets? _____ minutes. **Correct Answer: 5**.
- CRT_3_. In a lake, there is a patch of lily pads. Every day, the patch doubles in size. If it takes 48 days for the patch to cover the entire lake, how long would it take for the patch to cover half of the lake?_____ days. **Correct Answer: 47**.

As Frederick [34] points out, the peculiarity of the test lies on the fact that “… *The three items on the CRT are “easy” in the sense that their solution is easily understood when explained*, *yet reaching the correct answer often requires the suppression of an erroneous answer that springs “impulsively” to mind*.” (p. 27). In the test, these “erroneous answers” (100, 100 and 24, respectively) corresponds to the modal choices in our dataset [34]. Subjects’ test score corresponds to the number of correct answers (a number from 0 to 3).

Frederick [34] administered the CRT to 3,428 respondents, reporting moderate relations between CRT scores and several other psychological variables. CRT was related to time preference, risk seeking and cognitive measures (such as the Scholastic Achievement Test (SAT). Men score significantly higher than women on the CRT, and CRT scores are more tightly linked with risk preferences for men than for women. Although the CRT has a substantial correlation with cognitive ability, it is a unique predictor of performance on heuristics-and-biases tasks, as indicated by regression analyses [38]. CRT has likewise been shown to predict time and risk preferences, including preferences for options with higher expected value and resistance to logical fallacies [39, 40, 41]. Since CRT correlates with many behavioral domains of interest, we follow the cited literature by including a proxy of cognitive ability in our explanatory variables to identify this possible source of heterogeneity in the composition of the subject pool and its interaction with our treatment conditions.

Each subject was asked to maintain a regular sleep-wake cycle in the three days before the experiment: compliance was controlled by means of actigraphy (AMI MicroMini Motionlogger). Actigraphic mean sleep duration in the whole sample was 486±73 minutes in the three day before the post-sleep testing session and 466±69 minutes in the three days before the post-deprivation testing session (see below, Procedure section). Summary statistics of our subject pool are reported in the Supplementary Table (S1 Table).

### Procedure

After selection, subjects were randomly assigned to one of 8 groups, meaning that they participated in the experiment in groups of four (two females and two males). This study adopts a balanced design and consists of two laboratory sessions in which subjects are asked to perform the test battery (see below) under two conditions: after one night of undisturbed sleep at home and after one night of sleep deprivation in the laboratory. Therefore, all participants completed two testing sessions, which were both scheduled at around 10.00 a.m. There was a time interval of at least 1 week between the two experimental conditions. The order of conditions was counterbalanced between the eight groups: four groups (16 subjects) participated in the “sleep condition” first, while the other four groups participated in the “sleep deprivation condition” first.

All the subjects arrived at the laboratory around 19.00 hours to start the night of extended wakefulness Subjects were requested not to leave the room until the final testing session was completed. They were allowed to read, watch movies, study, listen to music, surf the internet and play games. Light snacks were permitted, while caffeinated beverages, chocolate, alcohol, and medications that can induce or contrast sleepiness were not allowed during the deprivation period. Time information was available. The lab was constantly illuminated by neon lamps (about 300 lux). Two experimenters (one female, one male) supervised the subjects throughout the night.

Subjects were tested in groups of four. They were seated in front of a 21-inch computer screen, at a distance of 50 cm. Printed instructions for each task were given to the participants; further explanations were given by one experimenter verbally, if requested. Each testing session, lasting about 15 min, included the RLP and DG tasks, which were always administered in the same (fixed) order. Before each testing session, all of the participants subjectively evaluated their alertness and sleepiness by means of a Visual Analog Scale (VAS) and the Karolinska Sleepiness Scale (KSS, see below).

As for the payment protocol, subjects were informed that one round across the 24 of each incentivized task (see below) would be drawn at random for payment at the end of each session. As is standard for multi-session experiments, subjects received their full winnings only at the end of the second session. Average payment was € 34 (SD = € 6).

### Tasks

**Incentivized Task 1: Random Lottery Pair (RLP)**. This task is based on 24 binary choices between lotteries involving four fixed monetary prizes (€ 0, € 5, € 10 and € 15). Subjects are asked to select their preferred lottery by clicking on the corresponding button (see Appendix B for more details on the experimental instructions and user interfaces). Lotteries were selected from Hey and Orme’s [32] original design. In the regressions of Table 1 the probability of selecting the “riskier lottery” within the pair (i.e., the lottery with greater variance) has been used as dependent variable for this task. As a measure of the payoffs’ position (dispersion), the mean (variance) of each lottery can be used as a proxy of the associated profitability (risk), respectively. As a matter of fact, mean and variance are the primitives for the classic utility function we use for our structural estimation [42].

**Table 1:** Regression analysis.

	Risky Choice	Egoism Index	Risk Aversion	Inequality Aversion
	*Logit RE*	*Tobit RE*	*Structural estimates*	
Deprivation	0.630**(0.309)	−0.260** (0.132)	−28.59** (15.01)	0.425(4.817)
Gender	0.266 (0.29)	−0.045 (0.187)	−14.255 (12.541)	3.702* (2.209)
Gender * Deprivation	−0.816*** (0.248)	−0.188* (0.111)	32.262*** (13.234)	−7.547*** (2.631)
CRT	−0.155 (0.124)	0.140 (0.080)	4.942 (5.662)	2.11*** (0.659)
CRT * Deprivation	0.288*** (0.106)	−0.094* (0.047)	−12.050** (6.151)	−1.670 (1.621)
Delta KSS	0.108 (0.086)	0.016 (0.054)	−3.75 (5.769)	0.475 (0.740)
Delta KSS * Deprivation	−0.139** (0.074)	0.049 (0.030)	5.906* (3.250)	−0.447 (0.693)
Delta VAS_AI	0.002 (0.002)	0.002 (0.001)	−0.123 (0.108)	0.043*** (0.015)
Delta VAS_AI * Deprivation	−0.0004 (0.001)	-0.002* (0.0008)	−0.006 (0.122)	−0.058** (0.028)
Constant	−0.497 (0.358)	1.139*** (0.230)	21.949 (21.896)	4.022*** (41.729)
	${\overset{-}{\chi }}_{\left(1\right)}^{2}$ = 61.47	${\overset{-}{\chi }}_{\left(1\right)}^{2}$ = 172.97		
	p = (0.000)^1^	p = (0.000)^1^		
Observations	1,536	1,216	3,072	3,072
Subjects	32	32	32	32

Notes: *standard errors in parentheses;*

* p< 0.*10*,

** p< 0.*05*,

*** p< 0.*01;*

*Estimates have been performed with Stata 13*.

^*1${\overset{-}{\chi }}_{\left(1\right)}^{2}$*^ *is an Lr test on the null hypothesis of no difference between the pooled estimator and the panel one*.

**Incentivized Task 2: Linear Dictator Game**. As regards social preferences, we employ a variation of the classic DG [33], in which the proposer (Dictator) decides how a sum of money is to be divided and the responder (Recipient) has no option but to accept the Dictator’s offer. In other words, both players always obtain what the proposer “dictates”. Game theory would expect that a rational self-interested Dictator would always offer the maximal amount of money to /himherself. However, in contrast to these theoretical expectations, empirical evidence demonstrates that Dictators share at least part of their money [43]. Therefore, the amount offered by the Dictator is presumed to reflect a pro-social, inequity-averse tendency driven by social norms and moral sensitivity, often termed as “altruistic fairness” [44].

In our version of the DG, subjects are matched in pairs, where one Dictator *(D)* and one Recipient *(R)*.*D* must choose a specific allocation, γ∈{0, .01, .02, …, 1} across 101 alternatives, where an allocation consists of a pair of monetary prizes, *x*(*γ*) = (*x*_*D*_(*γ*), *x*_*R*_(*γ*)), with, *x*(*γ*) = (*x*_*D*_(*γ*), *x*_*R*_(*γ*)). By moving the slider (i.e., by varying γ), the Dictator is varying the monetary prizes s/he and the recipient receive, along a pre-specified segment with extreme points with coordinates *x*(0) and *x*(1), respectively (which is why we refer to this protocol as a *Linear Dictator Game*). Monetary prizes for Dictators and Recipients are graphically displayed, with exact quantities reported on top of each colored bar. Dictators modify the allocation by moving the slider at the bottom of the screen. This process ends when the OK button is pressed. Participants are informed that the Recipient would be one of the participants to the following session to make each subject at ease with his/her decision.

Let $\theta =\frac{x_{D}\left(1\right)-x_{D}\left(0\right)}{x_{R}\left(1\right)-x_{R}\left(0\right)},x_{R}\left(1\right)\neq x_{R}\left(0\right),$ denote the exchange rate between the Dictator’s and the Recipient’s payoff across the Dictator's choice set. Across all 24 choices, we vary θ to ensure that Dictators are exposed to problems of different distributional types. Some problems have θ <0 (i.e., by increasing her/his own payoff, the Dictator lowers the Recipient’s payoff, as in standard Dictator Games); some problems, in turn, are characterized θ >0 (i.e., the Dictator can increase both players' payoff, at varying exchange rates); some problems have θ = 0 (with only the Recipient's payoff varying, while the Dictator’s prize stays constant); and some problems have θ = ∞ (where only the Dictator's payoff varies). These features of the distributional problem allow us to measure a wider variety of the Dictators’ distributional concerns, such as *efficiency seeking* [45] or *status seeking* [46], which Dictator Games cannot identify as they only cover the case of θ <0-both in the case of the “classic” Dictator Game [33] where θ = -1 but likewise in the case of the “generalized” Dictator Game used by Andreoni and Vesterlund [25].

An “egoism index”, *EgoIndex*, is calculated for this task by measuring the share of the Dictator’s available pie s/he allocates to him/herself (conditional of the specific round choice set):

$$\mathrm{EgoIndex}\left(\gamma \right)=\frac{x_{D}\left(\gamma \right)-\text{min}\left(x_{D}\left(0\right),x_{D}\left(1\right)\right)}{abs\left(x_{D}\left(1\right)-x_{D}\left(0\right)\right)}$$ (1)

In other words, *EgoIndex*(*γ*) = 1 (*EgoIndex*(γ) = 0) if the Dictator gives him/herself the maximum (minimum) prize available (regardless of what the Recipient obtains). EgoIndex has been used as dependent variable for this task in the panel data regressions.

Detailed experimental instructions of the two tasks are reported in the Supplementary material (S1 Methods).

**Subjective alertness and sleepiness evaluation**. At the beginning of each testing session, subjects were asked to self-rate their current status by means of a Visual Analog Scale (VAS) with respect to the following dimensions: tiredness, energy and concentration. Each subject was required to assess ‘how do you feel right now’ with respect to the above reported sensations by making a stroke with a pen on a 100 mm long line, between the extremes of “not at all” and “very much”.

Scores for the items tired (reverse scored), energetic and concentrated were added together to obtain the Alertness Index (AI), with higher scores indicating greater alertness.

Subjective sleepiness was estimated by each participant using the Karolinska Sleepiness Scale (KSS) [47] and scored on a scale ranging from 1 (very alert) to 9 (very sleepy).

Two differential sleepiness and alertness indices were computed (*DeltaKSS* and *DeltaVAS_AI*) for the regression analyses, by subtracting the sleep scores from the deprivation scores for each subject. Therefore, *DeltaKSS* and *DeltaVAS_AI* reflect differentials in subjective perception of sleepiness and alertness (respectively) after sleep deprivation.

## Results

We have identified below a set of variables that are shown to be relevant—particularly, when interacted with the treatment condition—to explain the behavior variability of the subjects in both tasks.

Following an established tradition in empirical microecenomic analysis [48] our estimation strategy first involves a panel regression approach which efficiently uses the information provided by our data, where individuals are repeatedly observed over time performing tasks of a similar nature. This approach allows us to control for time invariant individuals' unobserved characteristics that are likely to affect the relationships under scrutiny.

We estimate the following model in our panel regressions:

$$y_{it}=x_{i}\beta +v_{i}+\varepsilon_{it},$$ (2)

where *i* refers to individuals *i = 1* … *N*, *t* denotes time, with *t = 1* ….*T*, *y*_*it*_ is the dependent variable, $x_{i}=\left(1,x_{i}^{1},\ldots ,x_{i}^{K}\right)$ is the vector of explanatory variables; *v*_*i*_ are random individual effects i.i.d. N(0, $\sigma_{v}^{2}$), *ε*_*it*_ are idiosyncratic error terms i.i.d. N(0, $\sigma_{\varepsilon }^{2}$) independently of *v*_*i*_ and *β* = (*β*_0_,. *β*_1_, …, *β*_*K*_) is the vector of estimated parameters. Individual effects capture unobserved heterogeneity, i.e. differences in expected behavior that are not related to the observed differences in the explanatory variables.

The dependent variables *y*_*it*_ are, alternatively, the binary variable *Risky Choice* which takes value 1 if the subject *i* has chosen the “riskier” lottery at time *t* (zero otherwise) and the continuous variable *EgoIndex*(1) bounded in the interval [0, 1], respectively. In the first case, the first column of Table 1 reports the estimated coefficients of a panel Logit random-effect model, whereby the sign of estimated coefficients provides the direction of the impact that each explanatory variable has on the probability of choosing the riskier lottery. In the case of the latter, the second column of Table 1 reports the estimates of a Panel Tobit random-effect model whose coefficients reflects the nature of the effect of each explanatory variable on the variation of *EgoIndex*.

Since the main aim of this study is to consider the impact of sleep deprivation on individuals’ risk and inequality attitude, we include the treatment variable *Deprivation* in the model. The variable takes value 1 if the experimental task has been performed after a night of sleep deprivation and 0 if it has been conducted after a night of sleep. This regression coefficient directly shows the differential of the effect of such a trait on the dependent variable with respect to the excluded category. For example, a coefficient of the *Deprivation* variable which is significantly different from zero in the Logit regression suggests that sleep deprivation significantly affects the probability of making risky choices with respect to the sleep status (the excluded category). Moreover, if such a coefficient is significantly positive (negative), this means that deprivation yields an increase (reduction) in the probability of making risky choices.

In a similar fashion, we add the gender status to our specification by means of the binary variable *Gender*, positive for female, while the *CRT* variable represents the number of correct answers obtained in the Cognitive Reflection Test. Furthermore, we augment our specification with variables built on the basis of subjective measures of sleepiness and alertness (*KSS and VAS_AI*), which have been collected twice, under both treatment conditions. Such variables turn out to be highly correlated with the treatment condition, so that they are likely to induce collinearity problems if directly included in our specification. To avoid this problem, we decided to consider differences in subjective perceptions between the two different experimental statuses (precisely, the take under deprivation minus the take after sleep). Therefore *DeltaKSS* and *DeltaVAS_AI* reflects differentials in subjective perceptions on sleepiness and mood (respectively) after sleep deprivation and can be considered as proxies for subjective “sensitivity” to the change in the treatment conditions.

All variables have been interacted with the deprivation dummy in order to understand if their impact on the dependent variable does change according to treatment conditions. In Table 1, interaction variables are labeled as Gender * Deprivation, *CRT* *Deprivation, *DeltaKSS* *Deprivation, *DeltaVAS_AI* *Deprivation.

There is a caveat here. Panel regressions are very informative, since they allow the impact of our explanatory variables to be measured *simultaneously*. However, they neglect relevant features of the underlying economic decisions. Take, for example, the lottery task. When making their lottery choice, subjects are supposedly comparing the *risk* associated with each choice (well represented by the lottery’s variance) with its *expected return*: subjects are willing to bear a higher risk, if this is associated with higher expected rewards. By the same token, it is quite natural to assume that Dictators, when deciding whether to lower their own payoff in favor of the Recipient, may look at the distributional consequences (i.e., the induced inequality in the payoff distribution). Put differently, both our proxies *RiskyChoice* and *EgoIndex* do not identify precisely the economic trade-offs underlying both tasks. For this reason, the last two columns of Table 1 further report Maximum Likelihood estimates of a structural model in which subjects are assumed to maximize a standard “mean-variance” (random) utility specification, where the parameters associated with the variance have to be interpreted as a measure of subjects’ *risk* and *inequality* aversion, in the RLP andDG of Tasks 1 and 2, respectively [49]. The structural estimation of the variance parameters is conditioned to the same set of explicative variables as in the panel data regressions. In all cases, we selected a 5% level of significance to reject the null hypothesis.

As far as the *Risk Elicitation task* is concerned, the empirical specification of our choice model shows that subject *i’*s expected utility at time *t* (omitted) is assumed to depend on the mean (*μ_k_*) and the variance ($\sigma_{k}^{2}$) of the chosen lottery, *L*_*k*_, plus an i.i.d. idiosyncratic error term, ε, which has an extreme value distribution:

$$u_{i}\left(L_{k}\right)=\mu_{k}-\beta \sigma_{k}^{2}+\varepsilon .$$ (3)

A positive value for the parameter of interest β suggests that subjects are characterized by risk aversion.

For the DG we assume, again, that subject *i*’s expected utility at time *t* (omitted) depends on *i*’s monetary payoff, *x*_*D*_, and the mean squared error of the Dictator’s and Recipient’s payoff, $\sigma_{\gamma }^{2},$ associated with the Dictator’s choice, *x*(*γ*), as follows:

$$u_{i}\left(\gamma \right)=x_{D}-\beta \sigma^{2}\left(\gamma \right)+\varepsilon ,$$ (4)

With $\sigma^{2}\left(\gamma \right)=\left[\frac{{\left(x_{D}\left(\gamma \right)-\mu \left(\gamma \right)\right)}^{2}+{\left(x_{R}\left(\gamma \right)-\mu \left(\gamma \right)\right)}^{2}}{2}\right].$

In this framework, a positive β can be interpreted as a measure of subjects’ inequality aversion, as it lowers utility as the difference in payoffs between Dictator and Recipient increases.

Unconditional estimates (i.e., setting *β*_1_ = … = *β*_*K*_ = 0) of Equation (3) provide a positive and significant value for *β*_0_ of about 19.1 (std. err. 5.4, *p* = .0000), thus suggesting that subjects belonging to our sample are risk adverse. By the same token, unconditional estimates of Equation (4) show that observed subjects are inequality averse, *β*_0_ = 4.3, std. err. 1.01, *p* = .0000). However, in order to explore the impact of treatment conditions and personal characteristics on risk and inequality aversion, we condition estimates of our parameters of interest on the same covariates included in the Logit and Tobit regressions. The last two columns of Table 1 show estimates of Equations (3) and (4) where we let the estimated parameters depend on individual characteristics and on the experimental treatment.

We now refer to Supplementary Material (S2 Methods) for a short note on Structural estimates and panel regression techniques adopted in the analysis.

First note that the likelihood ratio tests reported at the bottom of Logit and Tobit regressions in Table 1 confirm that we are correctly applying a panel approach which duly takes into account the importance of panel level variance, since it shows that there is a significant difference between pooled Logit and Tobit estimators and panel ones.

Moreover, the estimated coefficients of the first two columns of Table 1 suggest that the experimental status affects subjects’ risk and inequality attitude since the *Deprivation* variable is significantly different from zero. Tests of joint significance on the *Deprivation* coefficient and its interactions lead us to reject the null hypothesis of no effects of treatment on our dependent variables. The sign of the coefficients of our panel data regressions suggest that sleep deprivation, on average, increases the probability of making risky choices and inhibits altruistic motives. Similar conclusions can be drawn when we analyze results obtained by estimating our structural model of individuals’ choice, (Equations 3 and 4), which suggests that individuals’ risk and inequality aversion are affected by the treatment condition in the same direction suggested by panel regressions. However, the impact of sleep deprivation on inequality aversion is poorly identified.

Average gender effects are negligible in all estimated models, whereas the interaction variable between gender and deprivation is statistically significant in both Logit and structural estimates, thus suggesting that women become more risk averse than men after sleep loss. By the same token, both Tobit and structural regressions indicate that women become more selfish than men in deprivation.

As far as the CRT is concerned, estimates show that it is not significantly different from zero in all models, while its interaction with the deprivation dummy is always significant. Hence, in the case of subjects who obtained higher scores in the CRT, deprivation makes choosing riskier lotteries more likely and tends to induce a more altruistic behavior (p<0.10), with respect to individuals with low CRT scores. Structural estimates confirm that higher CRT scores are associated to higher risk aversion, yet they do not provide conclusive evidence as far as inequality attitude is concerned.

Similar considerations hold for the variations in subjective measures of sleepiness and alertness. In particular, only the interaction variables are significant. Both panel regressions and structural estimates suggest that, for subjects characterized by a higher “treatment sensitivity”, deprivation enhances risk aversion and lowers inequality aversion, although the effect is not always significant.

## Discussion

Our results show that sleep deprivation affects the probability of making risky choices and modifies subjects’ altruistic fairness. We likewise observe that the effects of sleep loss on both behaviors are gender specific. In fact, sleep deprivation causes a *decrease* of risky choices in females and an *increase* of risky choices in males. Moreover, women become more selfish after sleep loss.

The differential effects of sleep loss on men and women’s risky behavior are particularly intriguing. The fact that men make riskier choices after sleep deprivation is consistent with earlier studies showing that sleep deprivation increase impulsive behavior and risk taking in men [50]. However, the decreased risky choices in women after sleep deprivation are less trivial and deserve a more careful scrutiny. Interestingly, Acheson et al. [17], using the Balloon Analogue Task, find decreased risky behavior after sleep loss in women, but not in men. In keeping with this result, a study evaluating the ability of men and women to predict automobile accident risk the day after having slept only 5 h and after consuming a moderate dose of alcohol, shows that women are more able to detect risks than men [51].

It has been suggested that gender differences in risk taking may be due to differences in subjects’ evaluations of outcomes or to the way probabilities are processed [52]. Women tend to be less sensitive to probability changes and likewise tend to underestimate large probabilities of gains to a higher degree than men do. In other words, women appear to be more pessimistic in the gain domain [52]. The combination of both effects results in significant gender differences in average probability weights in lotteries framed as investment decisions. Women’s relative insensitivity to probabilities combined with pessimism may indeed lead to higher risk aversion [52].

Therefore, the gender differences in risky choices observed in our study may be due to an altered evaluation of rewards during sleep deprivation. According to this notion, functional neuroimaging studies on risky decision-making during sleep deprivation have shown increased activation of brain areas involved in reward valuation [53]. Venkatraman et al. [2] observe after sleep loss, independent of gender, an elevated activation to gains in ventromedial prefrontal cortex and ventral striatum, but an attenuated activation of anterior insula following losses.

Gender-related differences in brain activity during risk-taking tasks have been indeed reported [14]. In particular, while executing the Iowa Gambling task, brain activity in men is lateralized almost exclusively to the right hemisphere, with men showing significantly more activation in the right lateral orbito-frontal cortex (OFC) [14]. By contrast, women have a poorer performance and a greater activation in the left dorsolateral prefrontal cortex (DLPFC), left medial frontal gyrus and temporal lobe during this task. These results are coherent with those recently reported by using fNIRS-based imaging during performance of a different task (the Balloon Analogue Risk Task), showing that females take less risks and have higher level of bilateral prefrontal cortical activity, while males take greater risks while showing lower levels of right PFC activity [54].

Thus, neuroimaging evidence indicates that the brain mechanisms engaged by men and women when solving the same decision making task are different. In general, this empirical evidence suggests that the effects of sleep deprivation on decision-making performance might likewise be different in women and men as it involves a different brain activity.

An optimal frontal lobe functionality depends on sleep and subtend crucial cognitive functions, such as distributional decisions. The fact that sleep deprivation has more notable influences on the frontal lobe in females than in males [16] can partially explain the gender differences observed in our study. Indeed, we found that, after sleep deprivation, only females make more selfish choices. The same effect is not evident in males, in which sleep deprivation might have less impact on the frontal lobe [16]. Here altruistic behavior has been assessed by means of the Linear Dictator Game. In this task, in which one subject decides about the division of a sum of money, fair offers conflict with monetary self-interest but conform to social motives and therefore might induce a conflict between what the proposer wants to do and what he or she ought to do [55]. The fair offers in the Social Preference Elicitation Task thus might primarily be motivated by altruistic motives. Functional imaging studies have reported the activation of posterior inferior frontal gyrus and anterior medial prefrontal cortex as neural correlates of human altruistic cooperativeness and related factors, such as empathy and interpersonal interaction [56, 57, 58, 59, 60, 61]. These activations may be gender specific. For example, young adult females show greater cooperativeness as well as larger regional gray matter volumes than males, particularly in the social-brain regions including bilateral posterior inferior frontal and left anterior medial prefrontal cortices [62]. On the behavioral side, females are more empathetic than males [63, 64, 65]. It is noteworthy that a recent study showed that sleep loss reduces the individual’s ability to be empathetic towards others [66]. Future research should address the question of possible gender differences in empathic behavior in response to sleep loss and their possible influence on altruistic cooperativeness.

Several studies have shown the existence of a relationship between cognitive ability and economic behavior [24, 34, 67], suggesting that individuals with high cognitive abilities seem to be more patient and more willing to gamble in the domain of gains [34, 41]. We here found that sleep deprivation peculiarly affects the individual and social economic behavior of subjects with higher cognitive reflection. In fact, sleep deprivation makes these subjects more likely to choose riskier lotteries and induce a more altruistic behavior. An interpretation of this effect in terms of disinhibition due to sleep deprivation seems to be the most plausible. We can assume that the people with high CRT scores, that in general perceive themselves as less impulsive and less preoccupied with their future [34], become more susceptible to making impulsive decisions when sleep deprived.

As a final caveat, it should be acknowledged that our results have been obtained using a sample of young healthy good sleepers, so that their generalization to the population as a whole, or to specific sub-populations (e.g., stock-exchange brokers, middle aged subjects) should be directly demonstrated by future studies. Another acknowledged limitation is related to the choice of a fixed order of task administration, which may have in principle influenced the results.

In conclusion, our results indicate a higher risk propensity of males after a night of sleep deprivation. By contrast, females react in a different manner, i.e. by reducing their risky choices. Similarly, the willingness to benefit others (or, conversely, the unwillingness to harm them), represented by altruism, decreases in sleep deprived females. Altogether, female’s reactions to sleep deprivation can be framed in an evolutionary context. The emotional, physical and cognitive effects of sleep loss could be interpreted by the participants as a potentially threatening situation. Then, if one is aware of her/his reduced cognitive abilities after a night of continuous wakefulness, it may be more adaptive to accept less risks and to be more selfish at others' expenses. These two effects do indeed coexist in our female subsample, and may both reflect a more conservative/defensive attitude, possibly developed as a protective reaction towards the offspring. According to the “offspring risk hypothesis” [68], women have a tendency to see greater risks than men because if they perceive more risks in the world, they will be more effective at keeping any offspring in their’s care safe. Consequently, both the increase in risk aversion and the decrease in inequality aversion in females after sleep deprivation may be interpreted as a defensive reaction implying the adoption of an overly conservative behavior. Such a behavior may have been selected by evolutionary processes because it is adaptive in the sense of the preservation of the species. Anyhow, these gender differences in social behaviors deserve to be further investigated, particularly in ecological settings (financial markets, medical, military, security and public safety settings).

Finally, our results further suggest the importance of assessing the effects of sleep loss on economic decision making in other contexts, when wakefulness and sleep occurs at inappropriate biological times. In fact the reduction of sleep time and the misalignment between circadian and wake-sleep physiology in shift-workers and in people traveling across multiple time zones (jet lag) leads to impaired cognitive performance, learning, emotional reactivity and safety [69, 70].

## Supporting Information

S1 Methods Experimental instructions. (DOCX)

S2 Methods Estimation Strategy. Structural estimates and panel regressions.
(DOCX)

S1 Table Summary statistics of our subject pool. (DOCX)
