# Supplementary material for: Gender Differences in Sleep Deprivation Effects on Risk and Inequality Aversion: Evidence from an Economic Experiment
Source: PLoS One. 2015 Mar 20;10(3):e0120029. doi: 10.1371/journal.pone.0120029 (PMC4368427; doi:10.1371/journal.pone.0120029)
Supplement: S1 Methods — (DOCX) [file pone.0120029.s001.docx]

Gender differences in sleep deprivation effects on risk and inequality aversion: Evidence from an economic experiment

**S2 Appendix. Experimental instructions (translated from Italian)**

**a) Risk Elicitation task: the Random Lottery Pair (RLP*)* (Task 1)**

In each of the 24 rounds of Task 1, we present you two lotteries and you will have to choose your favorite one. At the end of the experiment the server will determine randomly one out of the 24 rounds, and you will be paid the money that results from playing the lottery you selected. In each round, there will appear two lotteries on your screen. You will have to choose one.

Each lottery assigns different probabilities to win four prizes of € 0, € 5, € 10 and € 15, respectively. Each prize is associated with one color. This association between prizes and colors will hold for all 24 rounds in this phase. In the figure below there is an example of a lottery. If you choose the lottery on the LEFT, you can earn € 0 with a probability of 12.5%, € 5 with a probability of 75% or € 15 with probability 12.5%. If, on the contrary, you choose the lottery on the RIGHT, you can earn € 0 with a probability of 25%, € 5 with a probability of 50% and € 15 with a probability of 25%. In each round, you simply have to choose your favorite lottery by clicking with the mouse on the corresponding button.

It is important that you play all the 24 lotteries as if it was the one determining your payoff. This is due to the fact that after the experiment the computer will choose one out of the 24 rounds, and will play the lottery chosen by you in that round. In summary, the money you earn depends on the round chosen randomly by the computer and on the result of the lottery chosen by you in that round.^[[1]](#footnote-1)^


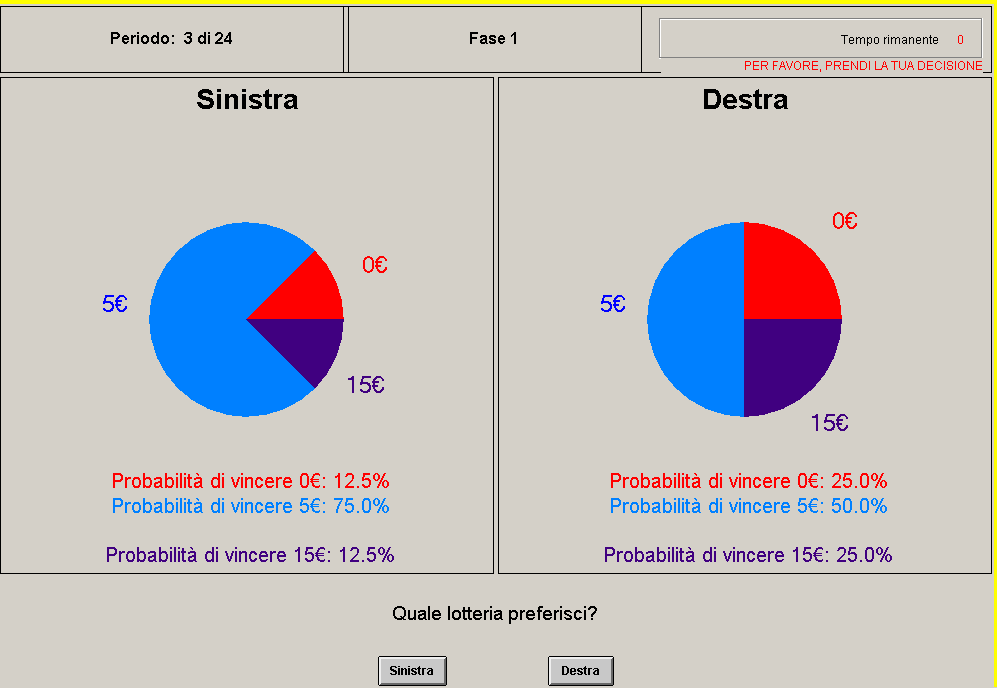


**Fig. a.** Task 1 user interface

**b) Social preference elicitation task (Task 2)**

In this phase, you will be randomly matched with a participant of the following experimental session (i.e., a person who is not in this room at this moment). For this task you will be defined as the CHOOSER, and the other person as the NON CHOOSER. By moving the slider, the CHOOSER has to choose across a set of possible money allocations, consisting of a certain amount of money for the CHOOSER and a certain amount of money for her/his randomly matched NON-CHOOSER.

In this stage the screen of the CHOOSER has a slider with two bars. The CHOOSER’s decision on where to place the slider has different consequences: it fixes the monetary gains for the CHOOSER and the paired NON-CHOOSER. As for Task 1, at the end of the experiment the computer will select one round for payment.


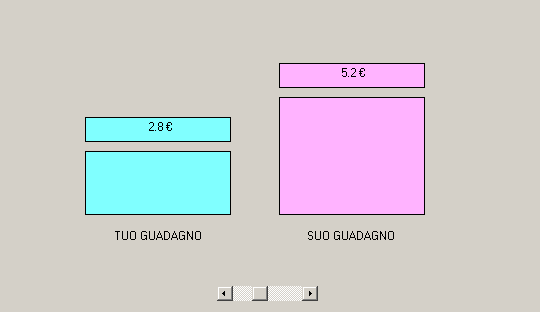


**Fig. b.** Task 2 user interface

- We ask you to move the slider back and forth several times before deciding where to stop and confirming your decision. You confirm your decision by clicking on the “CONFERMA” button. This will help you to get familiar with the entire set of possibilities at your disposal, before you decide on the allocation that you prefer.
- You will notice that moving the slider to the right *never decreases the amount of money for your matched* NON-CHOOSER, and typically increases the amount of money for the matched NON-CHOOSER. On the other hand, moving the slider to the right also changes the CHOOSER’s amount of money. Sometimes the amount of money for the CHOOSER will also increase; sometimes the amount of money for the CHOOSER will decrease; and sometimes the amount of money for the CHOOSER will not move at all. When the amounts for the CHOOSER and NON-CHOOSER change, they might not change at the same rate. It is important that the CHOOSER is aware of all the available possibilities before confirming a decision.
- The amount for the CHOOSER is drawn as a light blue bar on the left of the screen (“TUO GUADAGNO”), and the amount for the NON-CHOOSER is drawn as a pink bar on the right of the screen (“SUO GUADAGNO”). The two amounts of money shown on the screen are the two amounts that would be distributed if the CHOOSER confirmed at that very moment, and this decision was selected for payment. For example, if the CHOOSER confirmed the allocation in the above example, the CHOOSER would allocate € 2.8 to themselves and € 5.2 for the matched NON-CHOOSER.

To summarize, **your earnings in this stage are** determined by three things:

• by which round is chosen to be played out in the series of 24 rounds;

• by the set of allocations (selected by the computer) for that particular round; and

• by the decision of the CHOOSER in your matched pair.

1. We shall use the lottery pair reported in the instructions to explain what we exactly mean by “riskier” lottery in the paper, that is, the lottery characterized by the greater variance. In the RLP task, a lottery is a probability distribution over four monetary prizes: $L_{k}=\{x^{j};p_{k}^{j}\}$ , with *j*=1,…,4 and *k=*0,1, (*k*=0 for the Left lottery and *k*=1 for the Right lottery, respectively). In words, the four monetary prizes (€ 0, 5, 10, 15 in the experiment) are constant across lotteries, what changes are the probabilities. Each lottery is characterized by a *mean* (i.e., it’s expected value: $\mu_{k}\equiv E\left( L_{k} \right)=\sum_{j=1}^{4} x^{j}p_{k}^{j}$) and a *variance* (i.e., the expected value of the squared difference from the mean: $\sigma_{k}^{2}\equiv E\left( x^{j}-\mu_{k} \right)^{2}={\sum_{k=1}^{4} {(x}^{k}-\mu_{k})}^{2}p_{k}^{j}$). Simple calculations show that, in the case of the 2 lotteries of Figure B1, we have that $\mu_{0}=5.625; \mu_{1}=6.25; \sigma_{0}^{2}=15.234; \sigma_{1}^{2}=29.687.$This implies that, for the binary choice depicted in Figure B1, the “riskier lottery” corresponds to $L_{1}.$ [↑](#footnote-ref-1)
