# Supplementary material for: Gender Differences in Sleep Deprivation Effects on Risk and Inequality Aversion: Evidence from an Economic Experiment
Source: PLoS One. 2015 Mar 20;10(3):e0120029. doi: 10.1371/journal.pone.0120029 (PMC4368427; doi:10.1371/journal.pone.0120029)
Supplement: S2 Methods — Structural estimates and panel regressions. (DOCX) [file pone.0120029.s002.docx]

Gender differences in sleep deprivation effects on risk and inequality aversion: Evidence from an economic experiment

**S3 Appendix. Estimation Strategy**

Inference on attitudes towards risk and inequality can be derived by using different empirical approaches applied to experimental data. One possibility is to estimate a latent structural model of choice by using maximum likelihood (ML hereafter) methods in order to obtain the so-called “structural estimates” of the parameters of interest. Alternatively, experimental data can be analyzed by means of standard regression techniques which best take into account the nature of the data. In particular Logit and Tobit models suit this purpose given that our dependent variables are binary or bounded within a closed interval - [0,1], in our case, respectively.

**a) Structural estimates**

Our experimental design allows us to perform structural estimates of the parameters that provide information on subjects’ risk and inequality attitudes.

The risk attitude elicitation procedure adopted in this study is based on a series of Random Lottery Pairs (RLP) suggested by Hey and Orme (1994), who employed an RLP design to estimate individuals’ utility functions. The procedure implies a sequence of 24 binary choices over lotteries over four fixed monetary prizes. After choices are made, one of the pairs is randomly selected and played to determine monetary payoffs. We refer to Section 2 of the main text for a detailed description of the experimental task.

In the RLP design the probabilities of the two outcomes of each lottery are defined by the experimenter, so that the expected utility for a lottery $L_{k}$ is defined by adding up the utility values of each monetary payoff multiplied by their respective probabilities. For each lottery pair, it is possible to calculate an index, based on latent preferences, built on the difference between the expected values of the “right” and the “left” lottery, as displayed to subjects:

$\nabla U=\exp\left( u_{i}\left( L_{1} \right) \right)-\exp\left( u_{i}\left( L_{0} \right) \right)$. (1)

The index can be written in the form of a cumulative probability distribution function defined over differences in the expected utilities of the two lotteries as:

$\nabla U=\frac{\exp\left( u_{i}\left( L_{1} \right) \right).}{\exp\left( u_{i}\left( L_{0} \right) \right)+\exp\left( u_{i}\left( L_{1} \right) \right).}$ (2)

so that it maps the latent index values in the [0,1] interval and can be associated to actual choices by specifying that the “right” (left) lottery is chosen when the index gets value higher (lower) than 0.5. Expression (2) statistically links the difference in the expected utility of the two lotteries to the probability of the observed choice and is equivalent to $\wedge\left( {EU}_{r}-{EU}_{l} \right)$, where $\wedge\left( \cdot\right)$ is the logistic cumulative density function. On the basis of such hypothesis we can write the conditional log likelihood function for the choices made by subjects as:

$lnL\left( \theta,y,X \right)=\sum_{i} \left[ \left( ln\left( \nabla U \right)\left| y \right._{i}=1 \right) +\left( ln\left( 1-\nabla U \right) \right)\left| y \right._{i}=0 \right]$, (4)

where *X* is a vector of individual and treatment characteristics, *y* refers to the lottery choice and θ is a vector of parameters which stems from the assumptions made on the utility function.

In this study we assume a quadratic functional form for the utility function since, in this case, the expected utility can be defined just in terms of its mean and variance and it is consistent with the predictions implied by the “mean-variance” criterion on financial choices. Such criterion suggests that subjects prefer prospects with higher expected returns for a given level of risk (measured by the variance) and, at the same time, prefer prospects characterized by lower risk (lower variance) for a given level of expected returns. The curvature of the utility function give us information on individuals risk attitudes: in the presence of risk aversion (propensity), the second derivative of the utility function must be positive (negative); in the case of a quadratic utility function the sign of the second derivative is univocally determined by the sign of the coefficient of the parameter associated to the variance component of the empirical choice model (see equation 3 in the main text).

In similar fashion, we model subjects’ social preferences as elicited by means of a Dictator Game. Again we refer to Section 2 of the main text for a detailed description of the experimental task.

In particular, we assume that individuals care about others’ interests when choosing between possible allocations of a given monetary payoff and that they are characterized by different attitudes towards inequality as proxied by the dispersion of monetary payoffs between subjects involved in the experiment.

The empirical specification of our choice model (see equation 4 in the main text) is based on the assumption that subject *i* chooses allocation $\hat{k}$ if it maximizes its expected utility and the probability of such choice distributes according to a multinomial logit distribution:

$\Pr\left( y_{i}=\hat{k} \right)=\frac{\exp\left( u_{i}\left( L_{1} \right) \right).}{\exp\left( u_{i}\left( L_{0} \right) \right)+\exp\left( u_{i}\left( L_{1} \right) \right).}.$ (5)

The log-likelihood for the choice made by subject *i* can be derived by defining, for each individual, *d_ik_* = 1 if alternative *k* is chosen and 0 if not, for the *K*=2 possible outcomes. Then, for each *i*, one and only one of the *d_ik_*’s is 1:

$lnL\left( \theta,y,X \right)= \sum_{k=0}^{K} d_{ik}ln\left( Prob(y_{i}=k \right),$ (6)

where *X* is a vector of individual and treatment characteristics, *y* refers to the allocation choice and *θ* is a vector of parameters related to the assumptions made on the utility function.

In this framework, the curvature of the utility function give us information on individuals inequality attitudes: in the presence of inequality aversion (propensity) the second derivative of the utility function must be positive (negative). Again, the sign of the coefficient associated with the variance provides information on subjects inequality attitudes. Moreover, we let the parameter of interest, *β,* to depend on individual characteristics and on treatment conditions to explain the possible heterogeneity in behavior linked to individual characteristics and to the experimental status.

**b) Panel Regressions**

Experimental data can also be analyzed by means of statistical tools which allow us to investigate possible causal relationships among our variables of interest and explanatory variables that can be related with the experimental status and to individuals’ characteristics. In particular, panel regressions allow us to control for unobserved time invariant individual characteristics which are likely to affect the relationships under scrutiny.

**The Logit model**

Subjects’ risk attitude can be analyzed by estimating a regression where the dependent variable is a binary variable, *y_it_*, which takes on value 1 if the subject *i* has chosen the “riskier” lottery (i.e., the one the the highest variance within the pair) at time *t,* and zero otherwise.

Regression models for binary outcomes allow a researcher to explore how each explanatory variable (*x_it_*) affects the probability of the event occurring. In order to derive the binary regression model, we assume that we observe the realizations of an unobserved latent variable (*y**) which is related to the explanatory variables (*x’s*) through the equation:

$y\_it^*=x\_i \beta+\varepsilon\_it+v\_i$ (7)

where *i* shows the observation *i*=1…*N*, *t* denotes time, *t*=1….*T* , $x_{it}$ is a vector containing the *k* explanatory variables, $\varepsilon_{it}$ are idiosyncratic error terms, $v_{i}$ are individual specific effects which we assume to be random variables i.i.d. N(0,$\sigma_{v}^{2}$) independent from $\varepsilon_{it}$ and β is a vector containing the *k* parameters to be estimated. The latent variable is linked to the observed dichotomous variable *y_it_* so that:

$y_{it}=\left\{ \begin{matrix} 1 if y_{it}^{*}>0 \\ 0 otherwise \end{matrix} \right.$ (8)

Hence, when *y_it_** is positive, we observe *y_it_=1*, while negative or zero values of *y_it_** are observed as *y_it_=0*. For a given value of *x_it_* we can write:

$Pr=Pr \left( y_{it}=1 | x_{it} \right)=Pr\left( y_{it}^{*}\geq0 | x_{it} \right)=Pr\left( \varepsilon_{it}>-\left[ x_{i}\beta+v_{i} \right] | x_{it} \right)$ (9)

If F is the cumulative distribution function of $\varepsilon_{it}$ and is symmetric so that *1-F(-Z)=F(Z)*, we can write

$Pr=Pr \left( y_{it}=1 | x_{it} \right)=1-F\left[ -\left( x_{i}\beta+v_{i} \right) \right]=F\left[ \left( x_{it}\beta+v_{i} \right) \right]$ (10)

If we assume that $\varepsilon_{it}$ is distributed logistically with $Var\left( \varepsilon_{i} \right)={\pi^{2}}/3$, we obtain the binary Logit model which guarantees that as long as *x* increases the probability of observing *y_it_=1* increases but never steps outside the interval [0 1]; furthermore the relationship between *x’s* and such probability is non linear so that it approaches zero at slower rates when *x* decreases and approaches one at slower rates when *x* becomes large.

Given the above assumptions and assuming conditional independence, the joint probability of observing the *i*th observation *y_i_* can be written as:

$Pr\left( y_{i1}\ldots.y_{iT} | x_{i1}\ldots.x_{iT} \right)=\int_{-\infty}^{\infty} \frac{e^{{-v_{i}^{2}}/{2\sigma_{v}^{2}}}}{\sqrt{2\pi}\sigma_{v}} \left\{ \prod_{t=1}^{T} F\left( y_{it},x_{it}\beta+v_{i} \right) \right\}dv_{i}$ (11)

$F=\left\{ \begin{aligned} \frac{1}{1+exp(-{(x}_{it}\beta+v_{i}))} if y\neq0 \\ \frac{1}{1+exp(x_{it}\beta+v_{i})} otherwise \end{aligned} \right.$ (12)

The random effects Logit model is estimated by maximum likelihood (ML), which provides parameter estimates able to maximize the likelihood of generating the observed sample. With this method a *likelihood function* calculates how likely it is that we would observe the data we actually observe if a given set of parameter estimates were the true ones.

The panel level likelihood *l_i_* is

$l_{i}=\int_{-\infty}^{\infty} \frac{e^{{-v_{i}^{2}}/{2\sigma_{v}^{2}}}}{\sqrt{2\pi}\sigma_{v}} \left\{ \prod_{t=1}^{T} F\left( y_{it},x_{it}\beta+v_{i} \right) \right\}dv_{i}\equiv\int_{-\infty}^{\infty} g\left( y_{it},x_{it},v_{i} \right)dv_{i}$ (13)

In order to compute and maximize the log likelihood function we need to sum across individuals the logs of the panel-level likelihoods, *l_i_*. Even if there is no closed-form solution for integral (13), it can be solved numerically by approximating it with M-point Gauss-Hermite quadrature or with alternative quadrature methods. All estimates in this paper have been performed by way of the statistical package *Stata,* version 13 SE, by Stata Corporation.

**The Tobit model**

The outcomes of the Dictator Game can be analyzed by applying a Tobit model which duly takes into account the censored nature of the data, since our dependent variable*,* *EgoIndex,* is naturally bounded within the interval [0, 1].

Consider the linear regression model with panel-level random effects:

$y_{it}=x_{it}\beta+\varepsilon_{it}+v_{i}$ (14)

where *i* refers to the observation *i*=1…*N*, *t* denotes time, *t*=1,….*T*, $x_{it}$ is the vector containing the *k* explanatory variables; the random individual effects $v_{i}$ are i.i.d. N(0,$\sigma_{v}^{2}$) and $\varepsilon_{it}$ are idiosyncratic error terms i.i.d. N(0,$\sigma_{\varepsilon}^{2}$) independently of $v_{i}$, β is the vector of *k* parameters to be estimated.

The observed data, $y_{it}^{o}$ represent possibly censored versions of $y_{it}$. If they are left-censored, all that is known is that $y_{it}\leq y_{it}^{o}$. If they are right-censored, all that is known is that $y_{it}\geq y_{it}^{o}$ and if they are uncensored, $y_{it}=y_{it}^{o}$. If they are left (right)-censored, $y_{it}^{o}$ is determined by the left (right)-censoring variable and if they are uncensored $y_{it}^{o}$ it is determined by the explanatory variables.

Under the above assumptions the joint density (unconditional on *v_i_*) of the observed data for the *i*th observation can be written as:

$f\left( y_{i1}^{o}\ldots.y_{iT}^{o} | x_{i1}\ldots.x_{iT} \right)=\int_{-\infty}^{\infty} \frac{e^{{-v_{i}^{2}}/{2\sigma_{v}^{2}}}}{\sqrt{2\pi}\sigma_{v}} \left\{ \prod_{t=1}^{T} F\left( y_{it}^{o},x_{it}\beta+v_{i} \right) \right\}dv_{i}$ (15)

$F\left( y_{it}^{o},\Delta_{it} \right)=\left\{ \begin{aligned} {\sqrt{2\pi}\sigma_{\varepsilon}}^{-1} e^{{{-(y_{it}^{o}-\Delta_{it})}^{2}}/{2\sigma_{\varepsilon}^{2}}} ify_{it}^{o}\in C \\ \Phi\left( \frac{y_{it}^{o}-\Delta_{it}}{\sigma_{\varepsilon}} \right) if y_{it}^{o}\in L \\ 1-\Phi\left( \frac{y_{it}^{o}-\Delta_{it}}{\sigma_{\varepsilon}} \right) if y_{it}^{o}\in R \end{aligned} \right.$ (16)

Where C is the set of non-censored observations, L is the set of left-censored observations, R is the set of right-censored observations and $\phi(.)$ is the cumulative normal distribution.

The panel level likelihood is given by:

$l_{i}=\int_{-\infty}^{\infty} \frac{e^{{-v_{i}^{2}}/{2\sigma_{v}^{2}}}}{\sqrt{2\pi}\sigma_{v}} \left\{ \prod_{t=1}^{T} F\left( y_{it}^{o},x_{it}\beta+v_{i} \right) \right\}dv_{i}\equiv\int_{-\infty}^{\infty} g\left( y_{it}^{o},x_{it},v_{i} \right)dv_{i}$ (17)

As in the logit model, in order to compute ad maximize the log likelihood function we need to sum across individuals the logs of the panel-level likelihoods *l_i_*. Even if there is no closed-form solution for integral (17), it can be solved numerically by approximating it with M-point Gauss-Hermite quadrature or with alternative quadrature methods.
