# Supplementary material for: Gender Differences in Sleep Deprivation Effects on Risk and Inequality Aversion: Evidence from an Economic Experiment
Source: PLoS One. 2015 Mar 20;10(3):e0120029. doi: 10.1371/journal.pone.0120029 (PMC4368427; doi:10.1371/journal.pone.0120029)
Supplement: S1 Table — (DOCX) [file pone.0120029.s003.docx]

Gender differences in sleep deprivation effects on risk and inequality aversion: Evidence from an economic experiment

**Supplementary Table 1. Summary statistics of our subject pool.**

**Descriptive Statistics**

|  | Mean | St. Dev. | Min | Max |
| --- | --- | --- | --- | --- |
| Age | 24 | 2 | 20 | 28 |
| CRT | 0.94 | 1.02 | 0 | 3 |
| PSQI | 3.71 | 1.30 | 1 | 5 |
| BDI | 3.87 | 2.80 | 1 | 10 |
| ΔKSS | 4.2 | 1.79 | 0 | 7 |
| ΔVAS_AI | -133.6 | 73.54 | -250 | 9 |
| Risky Choice | 0.40 | 0.49 | 0 | 1 |
| Egoism Index | 0.80 | 0.31 | 0 | 1 |

**Notes**: 32 Subjects

CRT: number of right answers in the Cognitive Reflection Test

PSQI: score on the Pittsburgh Sleep Quality Index

BDI: score on the Beck Depression Inventory

ΔKSS: difference in subjective perception of alertness after sleep deprivation compared to baseline

ΔVAS_AI: difference in subjective perception of sleepiness after sleep deprivation compared to baseline

Risky Choice: number of Riskier Lotteries chosen

Egoism Index: score on the Egoism Index
